# Development of a Bioactive Polymeric Drug Eluting Coronary Stent Coating Using Electrospraying

**DOI:** 10.1007/s10439-019-02346-6

**Published:** 2019-08-22

**Authors:** C. M. McKittrick, M. J. Cardona, R. A. Black, C. McCormick

**Affiliations:** Department of Biomedical Engineering, University of Strathclyde, Graham Hills Building, 40 George Street, Glasgow, G1 1QE UK

**Keywords:** In-stent restenosis, Drug-eluting stent, Electrohydrodynamic spraying, Drug release, Stent-thrombosis

## Abstract

Drug-eluting stents are now routinely used in the treatment of acute coronary syndromes caused by coronary artery disease. Whilst the sustained release of anti-proliferative drugs from these devices has greatly reduced the need for repeat revascularisation procedures, this approach is not suitable for all patients and appears to delay regrowth of the endothelium, necessitating the use of prolonged dual anti-platelet therapy. Although the development of more advanced stent platforms and drug coatings has produced modest improvements in performance, these devices have not fully addressed the limitations experienced with their first-generation counterparts. In the present study, we developed a novel stent coating that provides controlled sirolimus release from a bioactive polymer (accelerate™ AT) that has previously been shown to support endothelial cell growth *in vitro*. A bespoke electrospray deposition process provided control over the coating thickness, surface roughness, drug load, and release kinetics. The resultant optimised coating combines rapid release of an anti-proliferative agent from a bioactive polymer coating that promotes re-endothelialisation, thereby offering potential protection against in-stent restenosis and thrombosis. This novel, dual-action coating therefore has significant therapeutic potential, with the enhanced control of drug load and release kinetics offered by electrospray deposition also opening up opportunities for more personalised treatment approaches. Further development and evaluation of these technologies *in vitro* and *in vivo* is therefore warranted.

**Electronic supplementary material:**

The online version of this article (10.1007/s10439-019-02346-6) contains supplementary material, which is available to authorized users.

## Introduction

Coronary artery disease (CAD) is caused by hardening and narrowing of the coronary arteries by atherosclerotic plaques, which results in reduced arterial blood flow and perfusion of the myocardium, leading to angina and localised ischaemia, which may eventually result in infarction.^25^ The most widely used treatment for CAD is now percutaneous coronary intervention (PCI), which involves permanent deployment of a stent into the target vessel, leading to immediate restoration of blood flow and symptomatic relief. Coronary stents are most commonly composed of a metal alloy and provide the radial strength required to maintain vessel patency. However, stenting induces the formation of neointimal tissue, characterised by inflammation and uncontrolled proliferation of smooth muscle cells, eventually causing thickening of the artery wall and late lumen loss.^14^ Drug-eluting stents (DES) target this hyperplastic response, termed in-stent restenosis (ISR), through the release of anti-proliferative drugs.^15^ This approach has proven to be very successful, with the incidence of ISR reduced from around 30–40% with bare metal stents (BMS) to less than 5% with DES.^5^ However, current DES are not as effective in high-risk groups, including diabetic patients and those with multi-vessel disease or complex atherosclerotic lesions.^5^ The drugs used on DES also inhibit endothelial cell proliferation, which may be an important factor in the delayed recovery of the endothelium that has been observed with DES.^17^ This delayed recovery exposes the sub-endothelial space and the stent surface to blood flow and leaves the vessel vulnerable to late and very late thrombosis.^31^ Although the extended use of dual anti-platelet therapy (DAPT) has helped ensure that stent thrombosis rates are now less than 1%, the high mortality associated with these events means that they continue to represent a significant clinical challenge.^4^ Consequently, much research in the last decade has focused on the development of stents that will inhibit restenosis without negatively impacting re-endothelialisation.

The drug release profile from first generation DES was controlled through the use of permanent polymer coatings. The observation that these devices may be associated with higher rates of thrombosis compared to BMS,^30^ served as a stimulus for the development of biodegradable polymer stent coatings. This alternative approach provides controlled drug delivery to the arterial tissue, before the polymer degrades to leave a BMS in the longer term. Nonetheless, recent clinical trials (COMPARE II and NEXT trials) found similar clinical outcomes for biodegradable coatings compared to durable polymer coated stents DES.^23^,^33^

A number of stent surface modification strategies have been investigated in order to provide coatings that accelerate re-endothelialisation of the lumen. Such approaches include modification of the polymer chemistry to mimic extra cellular matrix structures and attachment of molecules to promote cell adhesion.^34^ The attachment of bioactive molecules appears to be particularly promising. The Combo stent™ has anti-CD34 antibodies immobilised on its adluminal surface to capture circulating endothelial cells, and a sirolimus releasing coating on the abluminal side to inhibit smooth muscle cell proliferation. Results from the REMEDEE clinical trial indicated a regression of ISR and the absence of thrombosis at 2 year follow up.^19^ Furthermore, analysis of a small cohort at 60-days post stenting using optical coherence tomography, indicated that endothelial coverage was greater and neointima reduced in the group receiving the Combo stent™ when compared to an everolimus-eluting DES.^16^ However, the Combo stent™ has been unable to improve outcomes in some groups, with significantly worse outcomes in diabetic patients receiving insulin compared to non-insulin treated and non-diabetic individuals.^18^

Recent work has developed stent coatings with controlled and well-defined surface micro-topographies through the use of electrospray (ES) deposition techniques. By tuning the fabrication parameters, the resulting micro-topography of the polymer surface could be optimised.^10^ Ultrasonic atomisation is a very widely used method for applying stent coatings, with many manufacturers of clinically used DES making use of this technology.^8^ However, in-depth analysis of the topographical features of clinically used DES coatings produced in this way, has gone largely unreported in the literature. As the ultrasonic atomisation process can be prohibitively expensive for researchers carrying out pre-clinical development work, many such studies have instead used a dip coating technique, which, although convenient, has many drawbacks such as non-uniform coating, pooling of solution, bridging of stent struts and lack of control over coating thickness and surface topography.^1^

In this study, we developed a DES coating with a bioactive polymer that has been demonstrated to promote proliferation of human aortic endothelial cells and inhibit smooth muscle cell proliferation *in vitro,*^24^ and which has successfully completed a first-in-human trial,^21^ in which the stents were coated in polymer alone (in the absence of any drug). In the present study, we used a bespoke ES set-up to produce a series of accelerate™ AT-sirolimus coatings, with optimisation of the flow, electric field and distance across the electric field, providing control of coating thickness, drug load and roughness of the coating surface. We characterised sirolimus release from the polymer coatings by means of an *in*-*vitro* dissolution assay and used atomic force microscopy (AFM) to determine changes in coating thickness as well as surface roughness following drug release. Finally, we characterised endothelial cell growth on surfaces that were coated with polymer alone, in the absence of drug loading.

## Materials and Methods

### Coating Solution Preparation and Substrate Coating

Stent coatings were prepared using a polymer developed by Biomer Technology Ltd (Runcorn, UK): accelerate™ AT. The polymer is durable and biocompatible, with the composition and distribution of the functional groups present on the surface designed to mimic those of the extracellular matrix motif arginylglycylaspartic acid (RGD) *via* a combination of amine, carboxylic acid and hydroxyl groups in the correct density and proportion. The polymer and sirolimus (Cfm Oskar Tropitzsch, Marktredwitz, Germany) were dissolved in equal amounts in dimethylformamide (DMF) to a final concentration of 2% w/v and stored at 4 °C until used. A polymer only solution (2% w/v) was also prepared in DMF and stored at 4°C.

**Figure 1:**
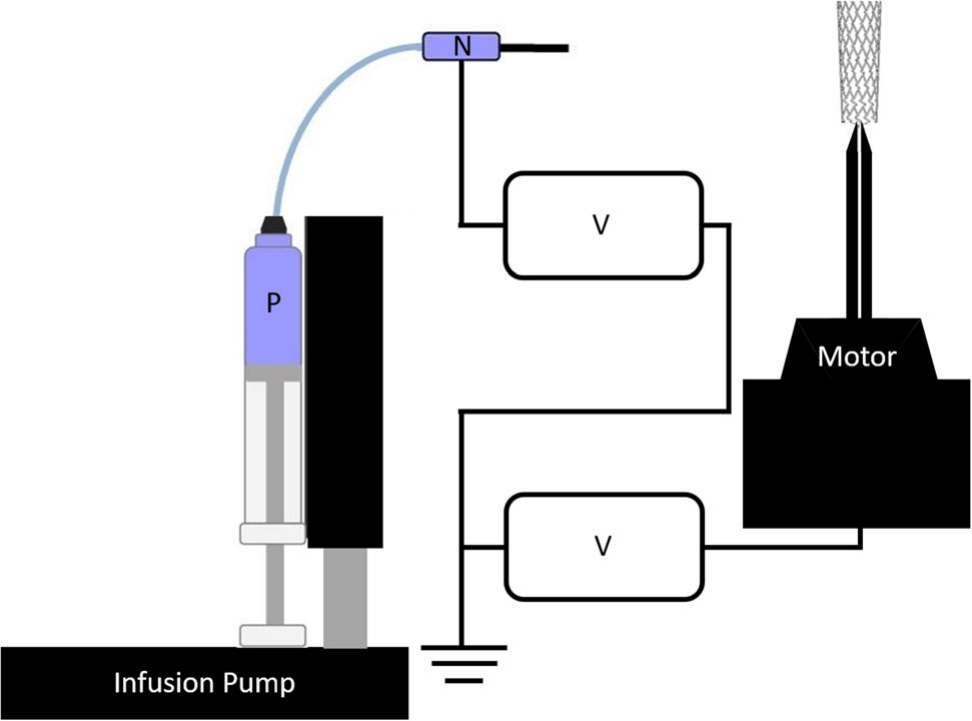
Schematic of electrospray apparatus. The setup includes high-voltage power supplies (*V*) connected to both collector (stent (shown) or coupon) and polymer solution (P). Polymer is driven to the needle at a rate of 2 mL/h by an infusion pump. The needle was set at 10 cm from the collector with the polymer being delivered to the collector across a potential difference of 20 kV. The collector is driven by a servo motor which rotates at a rate of 300 RPM. The motor remained stationary for coating of steel coupons.

Stainless steel 316L circular coupons (13 mm diameter, Goodfellow, Huntingdon, UK) and Multi-link™ coronary stents (8 mm × 3 mm, cobalt chromium alloy, Abbott Vascular, CA, USA) were coated using the 2% polymer/drug solution described above. The polymer was deposited by means of electrospraying (see Fig. 1), with the potential difference (20 kV) provided by two high voltage power supplies (Brandenburg, Alpha III, Dudley, UK). Polymer/drug solution was delivered by an infusion pump (Harvard Apparatus, PHD 2000, Kent, UK) at a fixed rate of 2 mL/h. Each coupon was electrosprayed for 5, 10, 20 or 30 min. The data collected from this initial investigation were then used to select stent coating times that would achieve a drug load and coating thickness comparable to that found on DES used in current clinical practice.^15^ Stents were therefore coated for periods of 10, 20 or 30 min. These coated stents were crimped onto a 15 mm-long balloon catheter (Conic Vascular, Santiago de Compostela, Spain) using a hand crimping tool. Additional circular coupons were either electrosprayed or dip-coated with the 2% polymer solution alone for use in endothelial cell viability assays.

### Sirolimus Loading and *In Vitro* Release

Stainless steel 316L circular coupons (*n* = 3) and stents (*n* = 3), coated as described above, were assessed for *in vitro* release of sirolimus, according to our previously detailed methodology.^22^ Briefly, stents were expanded to 3 mm diameter and fully immersed in release medium (phosphate buffered saline (0.01 M): ethanol (90:10), 1.5 mL). All incubations were carried out in sealed glass bijous under gyroscopic agitation at a rate of 20 rpm at 37 °C. The coated substrates were transferred to fresh medium at regular intervals, concluding at 28-days, at which point the coated substrates were immersed in methanol to strip residual drug. Samples were analysed by ultraviolet spectroscopy (Shimadzu Corporation, Japan) and absorbance at 278 nm recorded.

### Coating Characterisation

The surface roughness and thickness of coatings on stainless steel 316L circular coupons were quantified by Atomic Force Microscopy (AFM) (Oxford Instruments Asylum Research, MFP–3D-BIO, Santa Barbara, CA, USA). Surfaces were scanned in contact mode using ARiDrive–N01L tips. Representative scans were collected from three randomly-selected regions of interest from each coupon (*n* = 3) having a scan area of 50 *µ*m × 50 *µ*m at 0.5 Hz. Images were flattened in both *X* and *Y* axis to reduce alignment artefacts and their roughness expressed as the root mean square deviation from the mean line (R_RMS_). The thickness of coatings on coupons was established by physically removing the polymer at three randomly selected locations on each coupon (*n* = 3) and scanning at the interface between the polymer and metal surface. We approximated the thickness of the coating on the stent surfaces we produced. This was achieved by calculating the surface area of the stent (0.36 cm^2^) using the stent free area (87%) provided by the manufacturer and stent strut dimensions measured from scanning electron microscope images (see supplementary information). We used the sirolimus deposition rate measured from the coupons and stents from our experiments to calculate a ratio of drug deposition, per unit area (cm^2^). Scanning electron micrographs of stents were obtained using a table-top scanning electron microscope at an accelerating voltage of 15 kV (SEM, Hitachi TM1000, Krefeld, Germany).

### Cell Culture and Viability Assay

Endothelial cells were isolated from porcine pulmonary arteries, under local ethical approval, by gentle scraping of the lumen wall as described elsewhere.^12^ All reagents were purchased from Thermo Fisher Scientific, Paisley, UK. Cells were maintained in Medium 200 enriched with 2% low serum growth supplement and 1% penicillin/streptomycin and incubated at 37 °C. Cells were passaged when sub-confluent using TrypLE. Cells were used in experiments at passage 3–5 and were seeded at a density of 2 × 10^4^ cells/well onto polymer coated coupons, uncoated coupons and control wells in 24-well tissue culture plates in triplicate (*n* = 6). At 24-h intervals, culture medium was replaced with a 10% solution of alamarBlue™ and incubated for 1 h. The alamarBlue™ assay is able to detect redox activity in cells, which is directly related to cell viability.^27^ Aliquots (200 *µ*L) of the alamarBlue™ solution were then removed to a 96-well tissue culture plate and replaced with cell culture medium. Absorbance of the aliquots removed at each time point were then measured at 540 and 620 nm using a UV spectrophotometer (Multiskan GO, Thermo Fisher Scientific, Paisley, UK). The absorbance readings were processed according to the calculations detailed in the manufacturer’s instructions, and expressed in terms of the percentage reduction in alamarBlue™.

### Statistical Analysis

The data presented throughout this work are expressed as mean values ± standard deviation. Drug loading and coating thickness data were processed *via* linear regression and Pearson’s correlation coefficient in Excel (Microsoft, CA, USA). All further data analysis was conducted using Minitab software (Minitab Inc., PA, USA) and data were initially tested for normality using the Ryan–Joiner test. Comparison of coating thickness and roughness pre and post-elution was analysed by Students’ paired *t* test. Comparison of coating thickness, surface roughness and drug loads between coating times were analysed using one-way analysis of variance. Statistical significance of cell viability data was determined following repeated measures two-way analysis of variance, with post-hoc testing according to Tukey’s procedure at a 95% confidence level (*p* < 0.05).

## Results

### Polymer-Drug Deposition on Coupons

**Figure 2:**
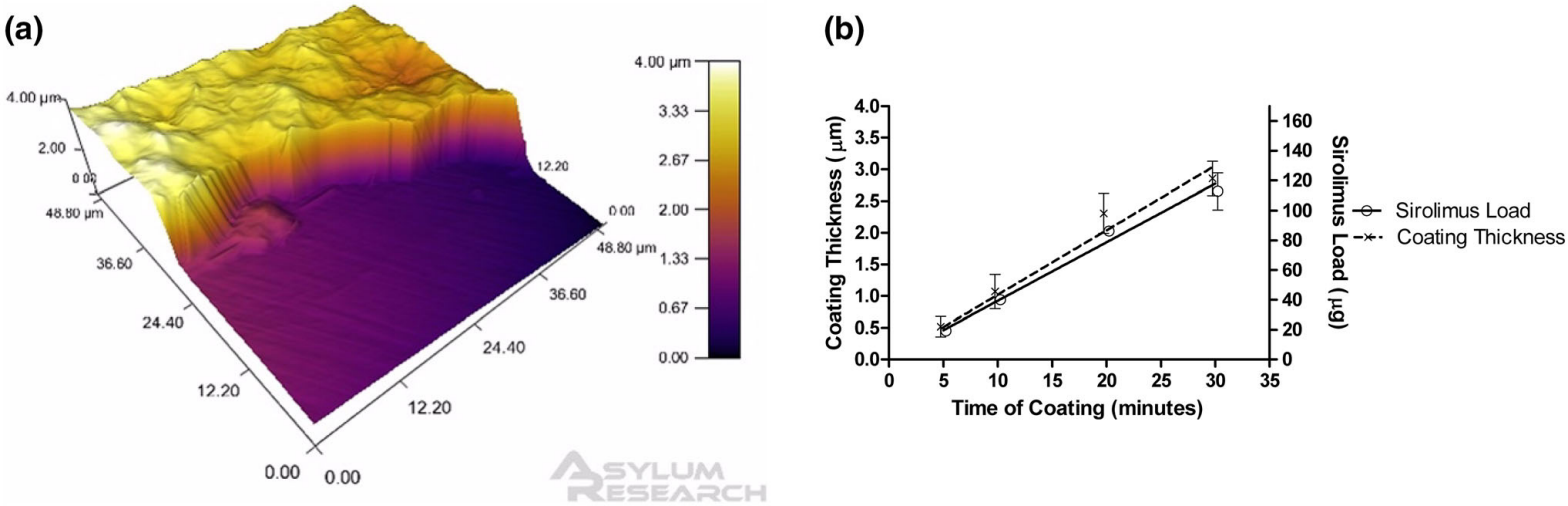
Atomic force microscope representations of drug-eluting polymer coatings on stainless steel coupons and polymer/drug deposition rates. Coating thickness was measured in three separate regions of interest in each of the coatings produced (*n* = 3). A representative atomic force micrograph of a 30-min polymer coating thickness is shown in (a). The relationship between both coating thickness (*R*^2^ = 0.97) and drug load (*R*^2^ = 0.99) with coating time were approximately linear (b) (*n* = 3).

We measured the thickness of the coating using AFM (Fig. 2a), and found a linear relationship between length of coating time and coating thickness (*R*^2^ = 0.97). The corresponding polymer-drug deposition rate was 102 nm/min for up to 30 min *(*Fig. 2b*)*.

Stripping of sirolimus from the coatings indicated that the mass of sirolimus loaded on to the surface was also time dependent. Increasing coating time resulted in a near linear increase of sirolimus load at a rate of approximately 3.4 *µ*g /min (*R*^2^ = 0.99) (Fig. 2b). Sirolimus was loaded onto the surface of the coupons as follows: 5-min coating (14.5 ± 3.25 *µ*g), 10-min coating (33.9 ± 4.61 *µ*g), 20-min coating (72.9 ± 8.34 *µ*g) and 30-min coating (100 ± 17.4 *µ*g).

### Polymer-Drug Deposition on Stents

**Figure 3:**
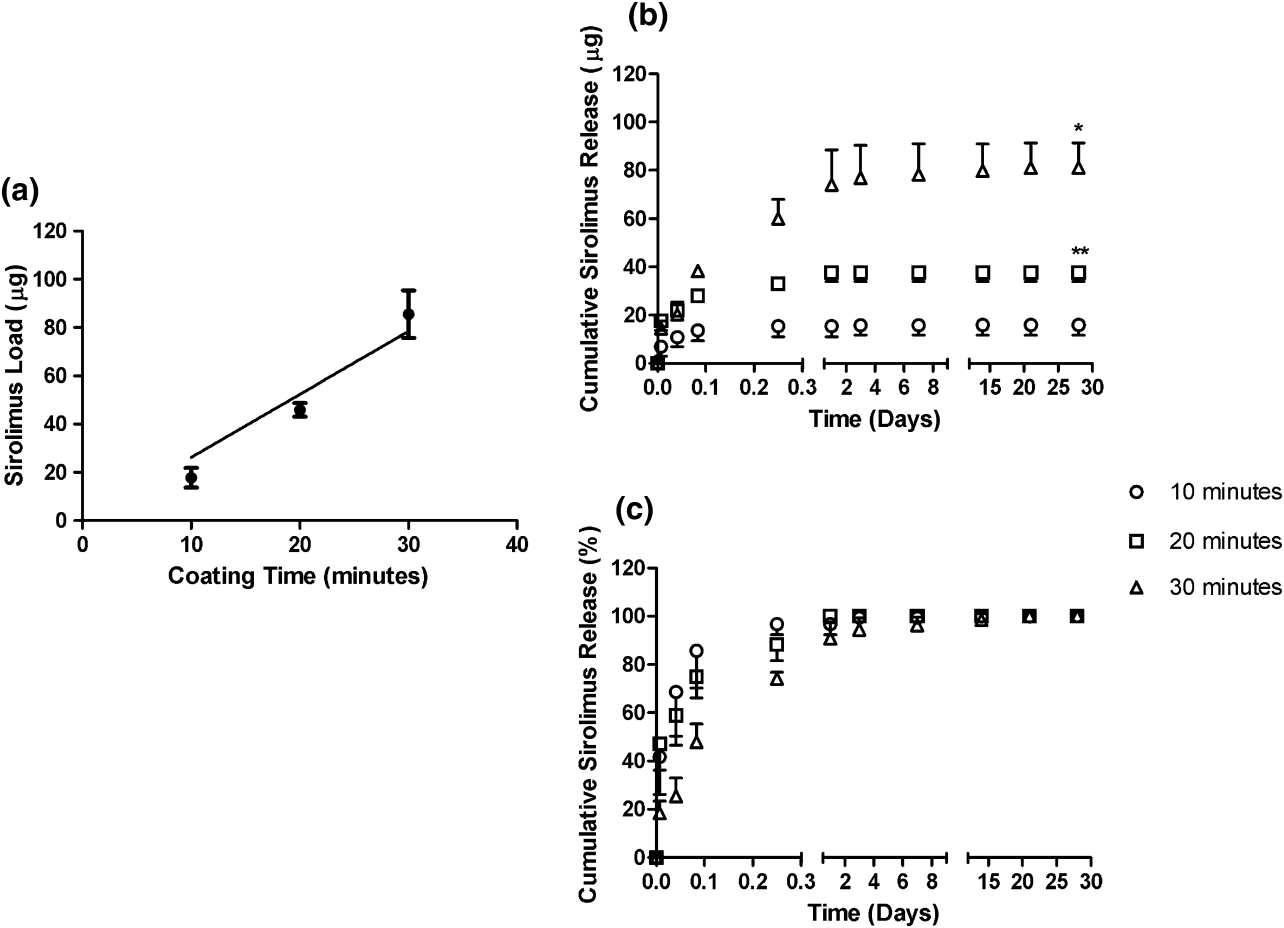
Coating deposition and sirolimus release from stents. Stents were coated using a 2% polymer: sirolimus solution for 10, 20 or 30 min (*n* = 3). Sirolimus was removed from coated stents and quantified using ultraviolet spectroscopy. Regression analysis indicated that sirolimus was deposited on to the stent surface at a rate of approximately 2.62 *µ*g/min (a). In-vitro release of sirolimus over a 28-day period indicated a significant increase in cumulative mass released with increasing coating time (*p < 0.05 30-min vs. 20-min coat and **p < 0.05 20-min coat vs. 10-min coat). Data were analysed by one-way ANOVA (b). Percentage mass released is shown in (c).

We estimated that drug was loaded on to stents as follows: 10-min coating, 17.8 ± 4.09 *µ*g, 20-min coating, 45.9 ± 2.85 *µ*g and 30-min coating 85.5 ± 9.76 *µ*g. Mean drug load values where plotted against time and this indicated that there was a strong positive correlation between coating time and mass of sirolimus deposited (*R*^2^=0.93), with sirolimus being deposited on to the surface at a rate of approximately 2.62 *µ*g/min (Fig. 3a). Thickness of the coating on stents was estimated using the deposition rate/cm^2^ and indicated that the coating thickness increased at a rate of approximately 286 nm/min. This calculation approximated the thickness of the coatings on the stents to be 2.86, 5.7 and 8.6 *µ*m on the 10- , 20- and 30-min coated stents, respectively.

The release of sirolimus *in vitro* over a four-week period, indicated that the total mass eluted by 10-min coated stents (15.9 ± 4.26 *µ*g) was significantly less than the 20-min coating (37.6 ± 3.67 *µ*g), which in turn was significantly less than the 30-min coating (81.1 ± 10.3 *µ*g) (all *p* < 0.05) (*n* = 3) (Fig. 3b). The release profiles varied for each of the coatings produced, although all of the coatings had released the majority of the drug after one day. The 10- and 20-min coatings released approximately half of the drug within 1-h , with a slower release for the 30-min stent coating, which did not reach 50% release until 6 h. Complete release was achieved by the end of the first day for the 20-min coating and by day 14 for the 10-min coating. Again, the 30-min coating release was slower, with 100% release not being recorded until day 21 of the experiment (Fig. 3c).

### Coating Characterisation and *In Vitro* Stability

**Figure 4:**
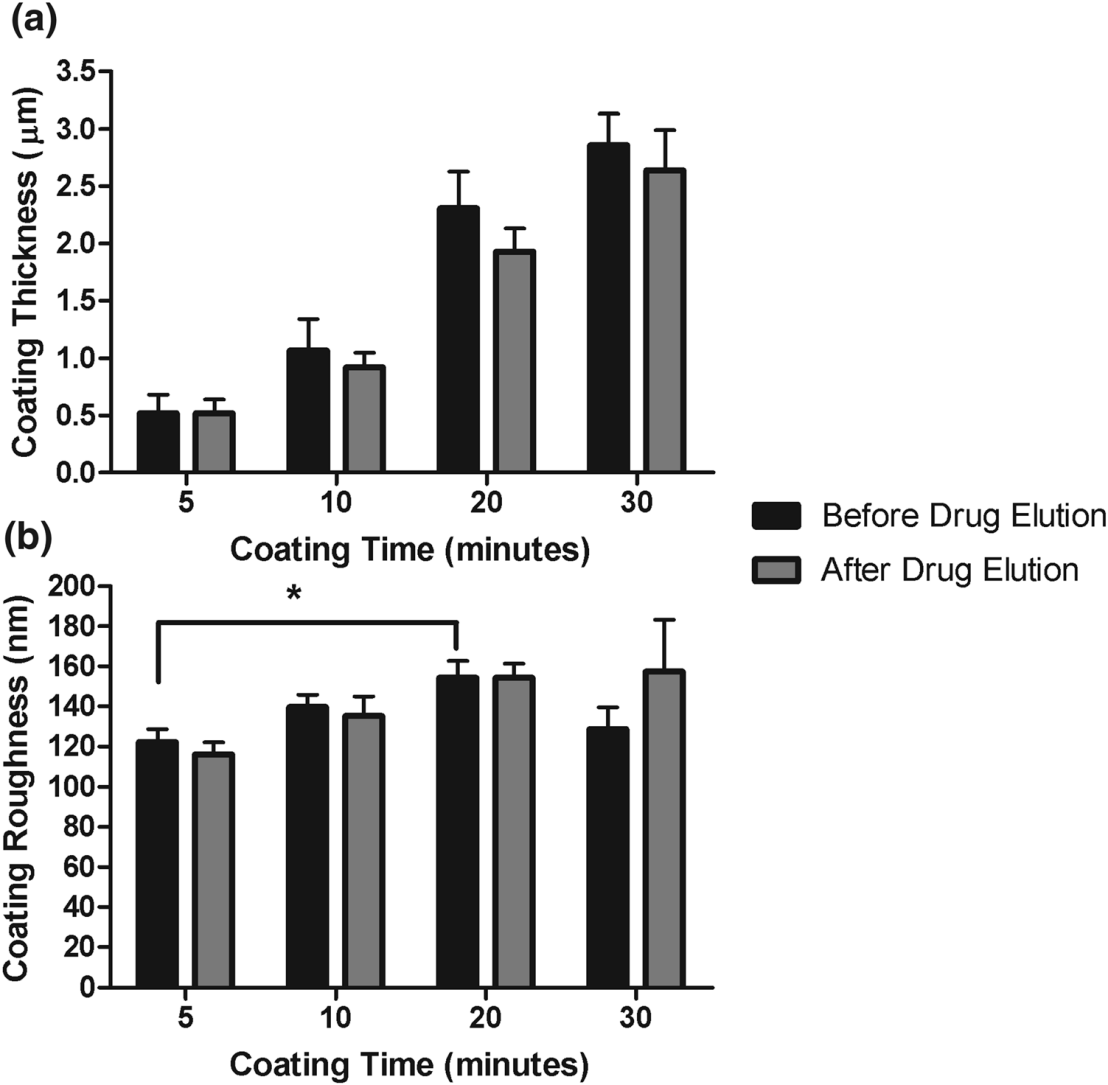
Thickness and roughness of electrosprayed drug-eluting coatings on steel coupons. Surface thickness was measured before and after removal of sirolimus and was shown to be unaffected by this process (a). Roughness of the surface was significantly increased on 20-min coated coupons when compared to 5-min coating (*p < 0.05) (b). Surface roughness was similar following 28-day removal of sirolimus for all coatings (b). Data were analysed by students paired t-test and one-way ANOVA.

The thickness of the coating on the coupons was unaffected by elution/removal of sirolimus, with no significant difference in thickness between pre-drug elution and post-drug elution coatings (*p *> 0.05) (Fig. 4a). However, the surface roughness was affected by the coating time, as we detected a significant increase in roughness values for the 20-min coating (154 ± 10.2 nm) when compared to the 5-min coating (122 ± 7.88 nm) (*p* < 0.05) (Fig. 4b). Nonetheless, coatings remained stable following drug elution as no differences were detected within any of the samples following the removal of sirolimus, further demonstrating the *in vitro* stability of the coating (Fig. 4b).

**Figure 5:**
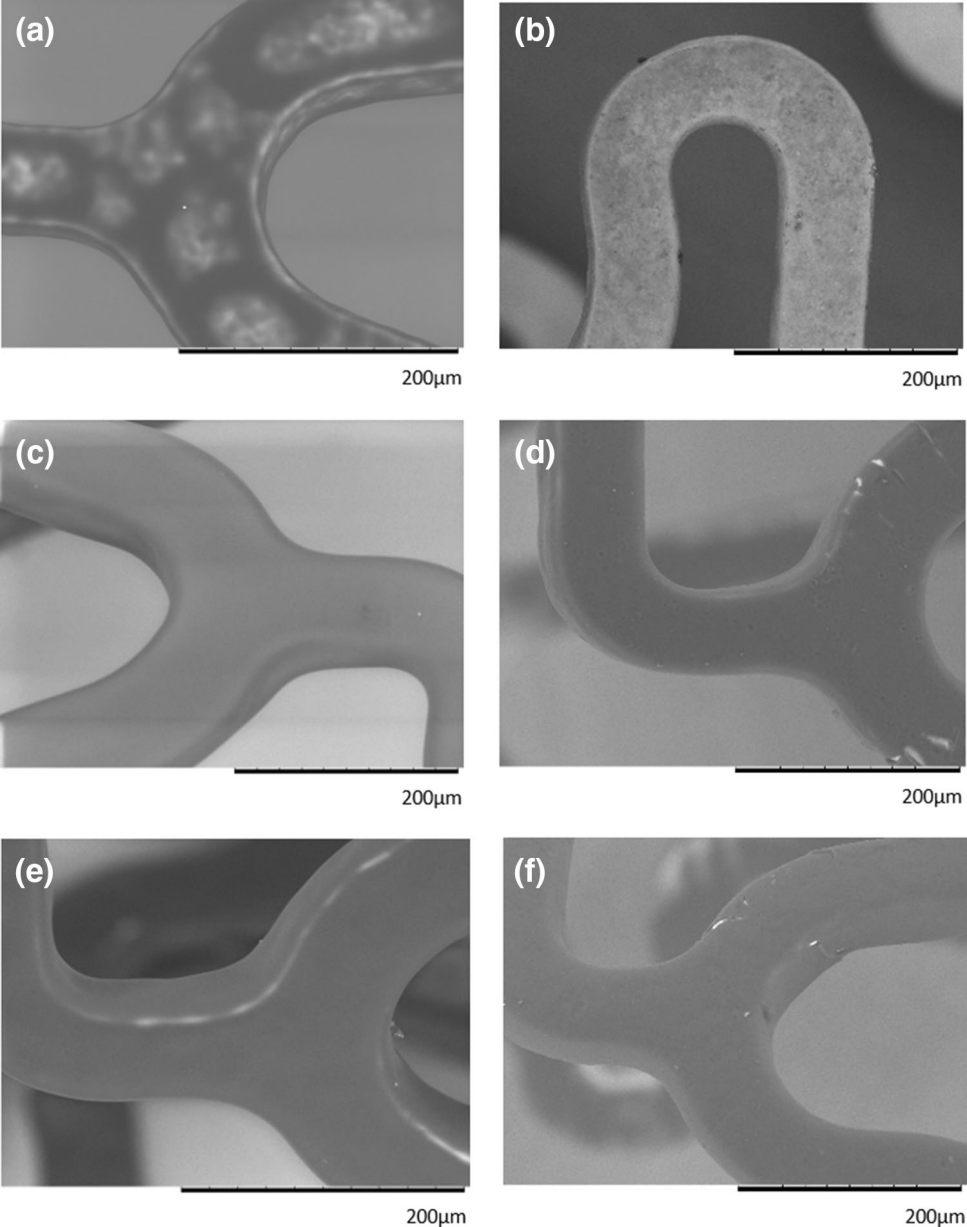
Scanning electron micrographs of electrospray coated drug-eluting stents. Stents were coated with a 2% polymer: sirolimus solution for 10, 20 or 30 min. Stents were imaged prior to any further manipulation. The 10-min coating appears heterogeneous with the stent surface still visible (a). Following 20 (c) and 30-min (e) of coating the surface appears uniform and free from imperfections. The stents were then crimped onto a balloon catheter, expanded and immersed in drug release medium for 28 days and then imaged again. The majority of the coating on the 10-min stent appears to have been removed during drug elution (b). Some minor cracking appeared within both the 20 (d) and 30-min (f) coatings.

To monitor uniformity of the polymer coating on stents, images were acquired before *in vitro* dissolution testing using SEM. The 10-min coatings were heterogeneous, with the metal stent surface clearly visible through the coating (Fig. 5a). However, the coating appeared more uniform when a 20 (Fig. 5b) or 30-min coating (Fig. 5c) was applied, with the stent surface completely covered in a homogenous coating. The coating also appeared to become thicker with time, as the edges appeared rounded and less sharp in the 30-min coating, compared with the 20-min coating. Following sirolimus removal, the 10-min coated stents appeared to have shed the majority of their coating, with the large patches that were visible prior to sirolimus removal no longer evident (Fig. 5d). Some cracking appeared around the edges of the struts of the 20 and 30-min coated struts exposing the metallic surface below (Figs. 5e and 5f).

### Endothelial Cell Viability Assay

**Figure 6:**
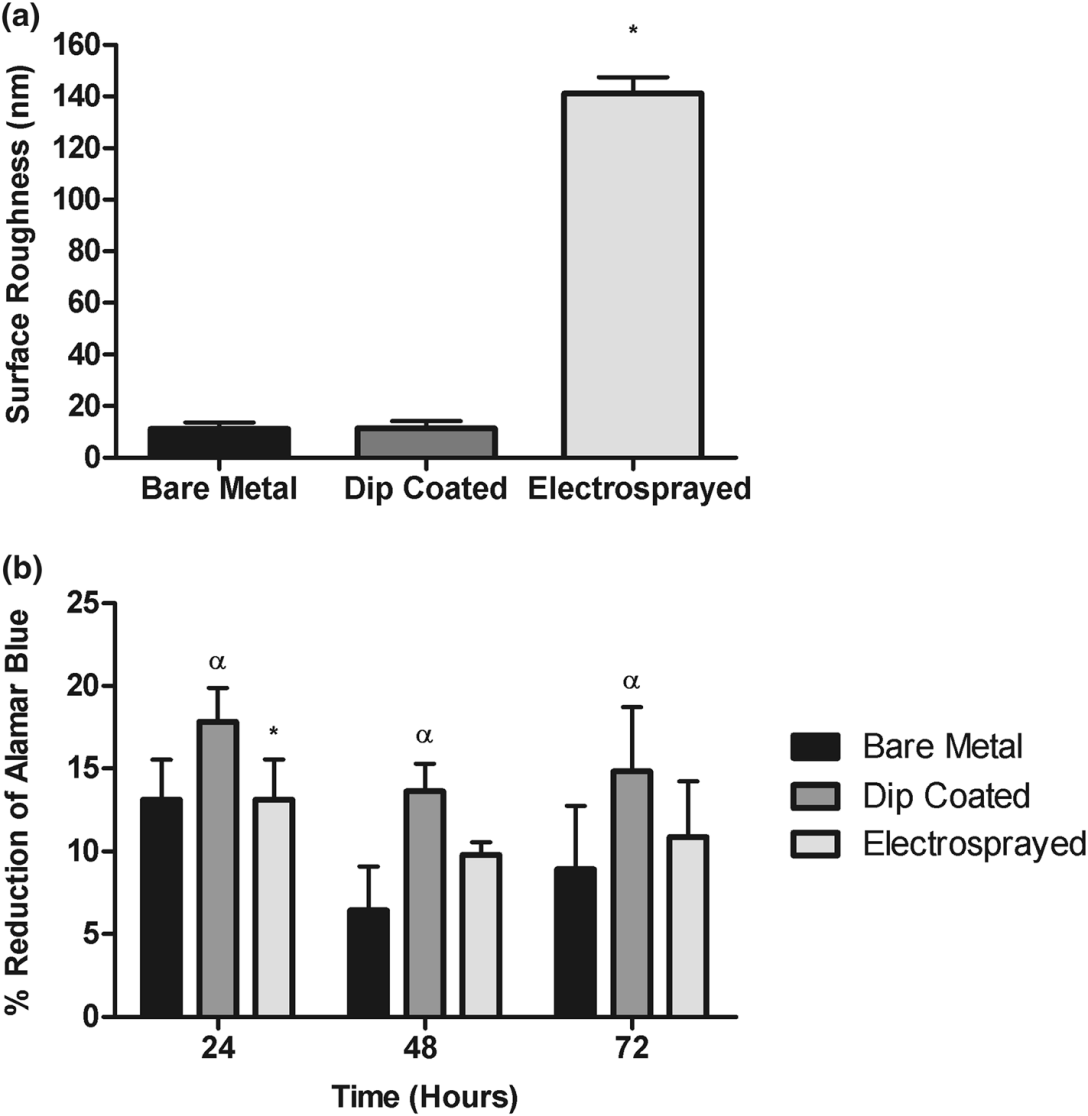
Viability of primary endothelial cells on drug-free electrospray coated and uncoated surfaces. Surface roughness on bare metal, dip-coated and electrosprayed polymer surfaces was measured by atomic force microscopy and indicated a significantly greater roughness value for electrosprayed surfaces, compared to bare metal and dip coated surfaces (*p < 0.05) (a). Cells were seeded at a density of 2 × 10^4^ cells/well onto polymer coated coupons, uncoated coupons and control wells in 24-well tissue culture plates in triplicate (*n* = 6). Cell viability was measured every 24 h for up to 72 h using an alamarBlue™ reduction assay (b). There was a significantly greater cellular viability on dip coated surfaces compared to bare metal at each time point, (*αp *< 0.05). At 24 h cell viability was greater on the electrosprayed surface when compared to the bare metal surface, (*p < 0.05). Cell viability was similar between the two polymer coated surfaces throughout. Data were analysed by 2-way repeated measures ANOVA with Tukey post-hoc comparisons.

The viability of primary porcine endothelial cells on coated and uncoated steel coupon surfaces was monitored at 24-h intervals for a 72-h period. We first measured the surface roughness of the substrates, which indicated that bare metal (11.1 ± 2.52 nm) and dip-coated (11.4 ± 2.74 nm) polymer surfaces were significantly smoother than the ES coatings (104 ± 1.78 nm) (*p* < 0.001) (Fig. 6a). Cell viability on the ES coated surfaces was significantly greater than the bare metal following 24 h in culture (Fig. 6b). Cell activity was also significantly greater on surfaces with a dip-coated polymer coating at each of the time points measured when compared to the bare metal surfaces (Fig. 6b). There were no significant differences between either polymer-coated surface over the entire 72-h period.

## Discussion

Although DES have revolutionised the treatment of CAD, the long-term efficacy of current coronary stent devices has not improved upon outcomes associated with coronary artery bypass grafting procedures.^6^ Furthermore, performance of DES remains limited among some patient groups and in the treatment of complex lesions,^25^ whilst prolonged dual anti-platelet therapy is still required in order to reduce the risk of stent thrombosis in many cases.^4^ Therefore, there are opportunities to improve on various aspects of stent design to improve device efficacy. In this work we investigated the application of a bioactive polymer coating and were able to control coating thickness, drug loading and surface roughness through optimisation of the ES process.

Using the ES process we were able to precisely control the mass of drug that was deposited on to the surface of the flat coupons and, to a lesser extent, the more complex geometry of actual stents. Importantly, the drug loads on the stent coatings we produced are in line with second generation DES with durable polymers coatings of the same length (8 mm), such as the PROMUS (38 *µ*g), Endeavour (84 *µ*g) and Resolute (59 *µ*g).^28^ The mass of drug loaded on to stents is an important determinant of DES efficacy, where a balance has to be met between achieving a therapeutic drug concentration within the arterial wall whilst simultaneously avoiding toxicity to both the smooth muscle cells and the endothelial cells.^3^ Findings from clinical trials have shown a dose-dependent effect of sirolimus, with increased sirolimus concentration significantly inhibiting lumen narrowing and incidence of restenosis at 6-month follow-up.^11^ Our more recent work using accelerate AT coated stents in a porcine coronary artery model also highlighted the importance of sirolimus concentration on DES efficacy, with a significant reduction in restenosis achieved with a higher dose of sirolimus after 28-days when compared with a lower dose.^22^

Drug release kinetics are also critical in achieving the balance between toxicity and therapeutic dose.^3^ The ideal release rate for a DES is still the subject of much debate, both slow sustained release and near zero-order kinetics having been proposed as a means of achieving optimal target receptor saturation.^3^,^29^ The coatings produced here displayed an initial burst release of the majority of the drug, with greater than 90% of drug released after one-day. Our recent modelling work using the accelerate™ AT polymer suggests that the mechanism behind this burst release profile is diffusive transport.^22^ Despite having a thicker coating and higher drug load, the 20-min stent had released 100% of drug by day one, whilst the 10 min-coated stent did not reach 100% release until day 14. A potential explanation for this is the difference in the surface roughness, with previous work indicating that rougher surfaces released drug faster when compared to planar surfaces, which is likely due to an increase in surface area.^20^ The 30-min coatings examined displayed a bi-phasic release profile similar to the 10-min coating, with an initial burst followed by a second, relatively slow phase. Whether this difference was directly related to the drug load or coating thickness, or both, remains to be seen. However, these findings warrant further investigation of the ES process and its utility in producing coatings with variation in drug load, coating thickness and surface roughness in order to identify the key parameters for further optimisation of the drug release profile. Further modification of the release kinetics could be achieved by addition of a drug-free polymer only top coat in order to slow release. Such a strategy was employed during the development of the first generation CYPHER DES, with a fast release device, which had similar release kinetics to the coatings we have produced, modified by the addition of a top coat which drastically slowed release. 32 Nonetheless, fast release polymer-free DES have demonstrated comparable clinical outcomes to existing, slow release polymer coated DES (BioFreedom trial) at 5-year follow up.^7^ Furthermore, these devices have been demonstrated to be safe and efficacious in high bleeding risk patients. 9 The release of the majority of drug in the initial burst phase of the DES shown here fits with the rationale behind such polymer free stents, although after the drug is released a bioactive polymer would remain rather than a bare metal surface.

It has been suggested that the thickness of the polymer coating impacts on the safety of the stent, with recent computational studies indicating that thicker coatings pose a greater risk of deformity.^13^ Such deformities in durable polymer coatings have the potential to induce thrombosis and increase ISR when implanted *in vivo.*^2^ The thickest coating that we examined was in the region of 8 *µ*m (30-min deposition time), which is below the range tested in the aforementioned study, but consistent with the typical coating thickness reported for currently used DES.^13^ While our coatings did show some evidence of minor deformities, we made this observation after they had been in release medium for 28-days following expansion under pressure. In the case of the 20 and 30-min coatings, some cracking appeared around the edge of the stent struts leading to some exposure of the underlying metal but there was no evidence of delamination. The magnitude of these areas of exposure is relatively small in comparison to the ‘craters’ that have been reportedly found in the coating of DES intended for clinical use.^2^ Other imperfections that have previously been reported with stent coatings, such as polymer webbing between struts, buckling and coating fragmentation, for example, were not evident in any of the coatings produced in the present study.^2^

Recent work by Guo *et al.*,^10^ demonstrated that polymer deposition on to coronary stents by means of ES can be fine-tuned to allow control over the polymer surface topography at a micro scale, which may allow for recapitulation of the topographical features of tissue and therefore promote integration of the device *in vivo.*^10^ We applied this method to produce coatings using a polymer that has been designed such that it mimics RGD. The RGD peptide sequence is found on proteins present in the basement membrane of native arteries, and is required for cell adhesion, which is a crucial step in cellular interaction with the substrate.^34^ Earlier work demonstrated that growth of primary human aortic endothelial cells was promoted on this material and proliferation of smooth muscle cells was not stimulated.^24^ The viability of cells attached to the surface of the accelerate™ AT coatings produced by electrospraying or dip coating was significantly greater when compared to stainless steel surfaces. This result notwithstanding, it remains unclear to what extent the modified surface impacts on the normal functioning of the endothelial cell layer. The endothelium plays an important role in the regulation of vascular function, not least the regulation of vascular tone and permeability and the prevention of thrombosis.^26^ To fully evaluate the impact of the accelerate polymer on the function of the endothelium will require future evaluation *in vivo* and *ex vivo*.

In conclusion, we were able to demonstrate the utility of the ES process for the production of drug-eluting polymer coatings for application on to coronary stents. Optimisation of the process allowed for a degree of control over a number of surface and coating characteristics, including drug load, coating thickness and surface roughness. The stents we produced are comparable to DES currently used in clinical practice, with similar drug loads and release kinetics, as well as coating thickness. Furthermore, we have shown that coatings produced using this ES process can accelerate the attachment of primary porcine endothelial cells to the surface. The novel dual-acting stent we produced has significant therapeutic potential to inhibit restenosis through rapid release of sirolimus in the short term, and promote vessel healing through the restoration of a confluent endothelial layer in the longer term by virtue of the bioactive properties of the polymer coating. That the roughness of the polymer coating remained largely unchanged after drug elution, providing a relatively smooth and stable substrate on which ECs have been shown to grow and adhere, and which is able to resist thrombosis in the short term and ISR in the longer term, is a further benefit with important implications for the success of this approach. Clearly further work is needed, but the findings of this *in vitro* study may help to make the case for further pre-clinical and clinical trials and also open up opportunities to investigate the impact of drug loads and release kinetics on DES performance using the controlled ES process.

## Electronic supplementary material

Below is the link to the electronic supplementary material.

Electronic supplementary material 1 (DOCX 18 kb)
