# Supplementary material for: Development of a Bioactive Polymeric Drug Eluting Coronary Stent Coating Using Electrospraying
Source: Ann Biomed Eng. 2019 Aug 22;48(1):271–81. doi: 10.1007/s10439-019-02346-6 (PMC6928095; doi:10.1007/s10439-019-02346-6)
Supplement: Supplementary file 1 — Electronic supplementary material 1 (DOCX 18 kb) [file 10439_2019_2346_MOESM1_ESM.docx]

Supplementary Information

The following procedure was used to estimate the surface area of the stents used which would ultimately be used to estimate the thickness of the stent coating.

| Complete stent length | 0.80 | cm |
| --- | --- | --- |
| Stent diameter | 0.30 | cm |
| Open cylinder surface area | 0.75 | cm^2^ |
| Stent free area (provided by manufacturer) | 84 | % |
| Stented Area | 16 | % |
| Outside area covered in stent | 0.12 | cm^2^ |
|  |  |  |
| Cross-sectional area of individual struts was measured to be rectangular.  Total stent surface area covered by polymer is therefore assumed as “3A”. | | |
| A  ½ A  Total stent surface area (3 x outside area) | 0.36 | cm^2^ |
| Using data collected of the total drug stripped, the drug load rate  per unit area per minute can be calculated. These are different  due to the inherent mechanism of electrospraying. | | |
| Stent drug load rate per cm^2^ per minute | 7.23 | ug/ cm^2^ minute |
| Disk drug load rate per cm^2^ per minute | 2.58 | ug/ cm^2^ minute |
|  |  |  |
| Assuming drug is uniformly distributed in the polymer for both stents and disks, the change in drug load rate can only be attributed to a change in coating thickness rate. | | |
|  |  |  |
| Factor of drug load (disks : stents) | 2.80 |  |
| Disk coating thickness rate (measured by AFM) | 102.00 | nm/min |
| Stent coating thickness rate (calculated) | 285.60 | nm/min |
